# Transfusion related acute lung injury presenting with acute dyspnoea: a case report

**DOI:** 10.1186/1752-1947-2-336

**Published:** 2008-10-28

**Authors:** Altaf Gauhar Haji, Shekhar Sharma, DK Vijaykumar, Jerry Paul

**Affiliations:** 1 Department of Surgical Oncology, Amrita Institute of Medical Sciences & Research Center, Ernakulam (682026), Kerala, India; 2 Department of Anaesthesia, Amrita Institute of Medical Sciences & Research Center, Ernakulam (682026), Kerala, India

## Abstract

**Introduction:**

Transfusion-related acute lung injury is emerging as a common cause of transfusion-related adverse events. However, awareness about this entity in the medical fraternity is low and it, consequently, remains a very under-reported and often an under-diagnosed complication of transfusion therapy.

**Case presentation:**

We report a case of a 46-year old woman who developed acute respiratory and hemodynamic instability following a single unit blood transfusion in the postoperative period. Investigation results were non-specific and a diagnosis of transfusion-related acute lung injury was made after excluding other possible causes of acute lung injury. She responded to symptomatic management with ventilatory and vasopressor support and recovered completely over the next 72 hours.

**Conclusion:**

The diagnosis of transfusion-related acute lung injury relies on excluding other causes of acute pulmonary edema following transfusion, such as sepsis, volume overload, and cardiogenic pulmonary edema. All plasma containing blood products have been implicated in transfusion-related acute lung injury, with the majority being linked to whole blood, packed red blood cells, platelets, and fresh-frozen plasma. The pathogenesis of transfusion-related acute lung injury may be explained by a "two-hit" hypothesis, involving priming of the inflammatory machinery and then activation of this primed mechanism. Treatment is supportive, with prognosis being substantially better than for most other causes of acute lung injury.

## Introduction

Transfusion-related acute lung injury (TRALI) is a frequently misdiagnosed, yet potentially fatal reaction following transfusion of blood products. There is much confusion in the literature regarding this entity because until recently, there was no uniform nomenclature, definition or diagnostic features described in relation to it.

We describe a case report of TRALI, not because it is infrequent, unique or has never been described before, but to familiarize our colleagues with it. The intention of this article is to compile available information to educate ourselves to a potentially preventable life-threatening condition and the current guidelines for its management.

## Case presentation

**Figure 1:**
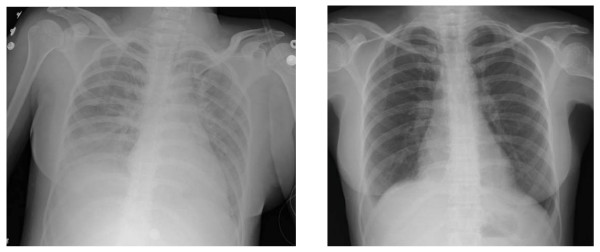
Chest X-ray findings (a) at the time of acute symptoms and (b) after weaning from the ventilator.

**Figure 2:**
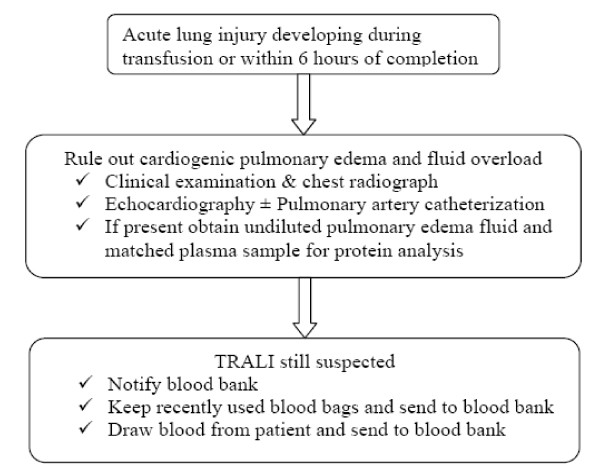
Flow chart to evaluate a case of acute lung injury within 6 hours of transfusion.

A 46-year old woman of Indian origin complained of breathlessness along with chest discomfort in the ICU, where she was recuperating from a laparotomy for ovarian malignancy. Her complaints had started within 20 to 25 minutes of completion of a transfusion of a single unit of packed red blood cells (PRBC). Rapid clinical deterioration was noted with a falling oxygen saturation (<80%), hypotension (systolic BP of <80 mmHg), tachycardia (>140/minute), tachypnea (>30/minute), and mild fever (100°F). An urgent chest X-ray was ordered and showed extensive bilateral pulmonary infiltrates (Figure 1). Invasive monitoring was initiated and a panel of investigations was ordered immediately (Table 1). Hemodynamic parameters progressively worsened with onset of respiratory distress. In the setting of a deteriorating clinical condition, the patient was supported with mechanical ventilation using a positive end expiratory pressure (PEEP) of 10 mmHg along with multi-agent hemodynamic support (dopamine, dobutamine and noradrenaline). Over a period of 72 hours, the patient responded to symptomatic measures. Her hemodynamic parameters improved and the vasopressor support could be withdrawn after 48 hours. However, recovery from the pulmonary insult was slower. X-ray showed clearance of pulmonary infiltrates after 72 hours of ventilator support and weaning was possible only after that (figure 2).

**Table 1:** Summary of immediate investigations done at the time of acute symptoms

S. No.	Investigation	Parameters	Value	Comments
1.	ABG	pH	7.447	Measured with O_2 _on flow at a rate of 10 liters/minute through nasal prongs
			
		pCO_2_	23.8	
			
		pO_2_	30.7	
			
		SpO_2_	62.2	
			
		Hb	10.4	
			
		Hct	32	

2.	Invasive hemodynamic monitoring	Arterial BP	85/48	Invasive monitoring instituted in view of the deteriorating hemodynamic status
			
		CVP	12	

3.	ECG	Normal sinus tachycardia (HR 144/minute) with no evidence of ischemic changes		

4.	ECHO	Normal study with no evidence of RWMA, normal LA parameters, LVEF 55%		

5.	Chest X-ray	Bilateral extensive pulmonary infiltrates, no effusion		

6.	Troponin T	-	0.352	NR: 0–0.2 ng/ml

7.	CK – MB	-	47	NR: 0–23 IU/ml

The initial differential diagnosis was between transfusion mismatch, myocardial infarction, pulmonary embolism and fluid overload. Absence of typical clinical features of a cross-match reaction such as bronchospasm, rashes, hemoglobinuria, renal shutdown, or falling hemoglobin levels, along with a negative recheck for cross-match reaction in the blood bank lab ruled out a mismatched transfusion. Fluid overload was ruled out by a normal central venous pressure (CVP) and normal echocardiogram (ECHO). A normal electrocardiogram (ECG), normal ECHO and near-normal cardiac enzymes ruled out the possibility of an acute myocardial ischemic event. Pulmonary embolism was excluded from the differential diagnosis on the basis of bilateral extensive pulmonary infiltrates, normal D-dimer values and no clinical evidence of deep vein thrombosis.

In this clinical setting, a possibility of TRALI was raised. The patient's clinical features, course of events, and response of the acute episode to supportive management, were all supportive of this diagnosis.

The patient eventually recovered completely from this acute pulmonary insult over the course of the next few days and was discharged from hospital care by the 10th postoperative day.

## Discussion

TRALI is defined as non-cardiogenic pulmonary edema temporally related to the transfusion of blood products [1,2]. The current definition of TRALI (see below), by consensus panel, includes two components, the first being Acute Lung Injury (ALI), and the second being absence of features of ALI before transfusion and its onset in temporal relation to transfusion of a blood product [2].

Criteria for ALI (AECC guidelines 1994; Toy et al., 2005 [2])

1. Timing: acute onset

2. Pulmonary artery occlusion pressure ≤ 18 mmHg when measured or lack of clinical evidence of left atrial hypertension

3. Chest radiograph: Bilateral infiltrates seen on frontal chest radiograph

4. Hypoxemia: Ratio of PaO_2_/FiO_2 _≤ 300 mmHg regardless of the positive end-expiratory pressure level, or oxygen saturation of ≤ 90% on room air.

In addition, for TRALI

1. Onset within 6 hours of transfusion of blood products

2. No pre-existing ALI before transfusion

3. TRALI still possible if another ALI risk is present

Massive transfusion should not exclude the possibility of TRALI

In 1951, Barnard described the first case of fatal pulmonary edema following transfusion therapy [3].

Popovsky is credited with having coined the term TRALI in 1983 to refer to non-cardiogenic pulmonary edema complicating transfusion therapy, when he reported a case series of 36 patients over a period of 3 years [4].

With the reduction of clerical errors and with more effective screening and prevention of the transmission of infectious agents, TRALI has surpassed hemolytic reactions as the leading cause of transfusion-related mortality in developed countries. It is, thus, now emerging as one of the most common serious complications of blood transfusion [5]. The published incidence of TRALI ranges from 0.02% to 0.05% per blood product unit transfused and from 0.08% to 0.16% per patient who received a transfusion [6]. The true incidence of TRALI is not known because there is significant under-reporting of cases [7].

Confusion surrounding TRALI is due to the numerous eponyms that have been used in the past to refer to this clinical entity. The syndrome had previously been referred to as *pulmonary hypersensitivity reaction*, *allergic pulmonary edema*, *non-cardiogenic pulmonary edema *and *pulmonary leukoagglutinin reaction *[7]. Consequently, the disease entity is still under-recognized and under-reported for a multitude of reasons, which vary from a lack of precise definition, misdiagnosis, to lack of awareness.

Almost any blood component containing about 50 ml or more of plasma is implicated; use of red blood cells (RBCs), and pooled platelets from several donors seems to have a particularly high risk [5,6,8,9]. Rarely, cryoprecipitate, intravenous immunoglobulin, and stem cell preparations have been implicated and it does not seem to occur with washed red blood cells [10]. Interestingly, the incidence is least with Fresh Frozen Plasma (FFP), and maximal with platelet concentrates.

Symptoms of TRALI usually appear within 2 to 6 hours from initiation of transfusion, but cases of presumed TRALI have been described up to 48 hours after transfusion [11]. Clinically, the patient presents with features of acute onset respiratory and hemodynamic complications in the absence of features of circulatory overload such as dyspnea, tachypnea, frothy sputum, fever, hypotension, or, much more rarely, hypertension [11].

The exact etiology of TRALI is unknown, but two distinct mechanisms have been suggested. The traditional theory proposes an antibody-mediated reaction between recipient granulocytes and anti-granulocyte antibodies from donors who were sensitized during pregnancy (multiparous women) or by previous transfusion [4,5].

Recently, an alternative mechanism has been suggested, implicating pro-inflammatory molecules, predominantly lipid products of cell degradation, known to accumulate during storage of cellular blood products [12].

Both models are based on a two-hit hypothesis wherein a first hit is required as an initial priming event followed by a second initiator event. Of note, the two hypotheses of TRALI pathogenesis are not mutually exclusive and may even act synergistically with underlying patient factors to produce acute lung injury.

The first insult (first hit) consists of priming and adherence of neutrophils to the pulmonary endothelium. Candidate conditions for producing the first insult in TRALI include surgery, sepsis, trauma, massive transfusions, hematologic malignancies, cardiac surgeries, induction chemotherapy and cardiopulmonary bypass [1].

The second insult (second hit) activates these primed neutrophils, resulting in the release of reactive oxygen species that cause capillary leak and pulmonary edema [13]. For the second hit, parity of the blood donor, relationship to the blood donor, and the age of the blood products can all be potential risk factors [1].

Although the antibody theory remains more widely accepted and published, in some cases, there is definite evidence of biologically active lipids in the etiogenesis of TRALI.

The first step in the management of TRALI is to make a correct diagnosis. This requires a high index of clinical suspicion and awareness about this condition in the event of any adverse episode temporally related to blood transfusion to diagnose and treat this condition effectively. Figure 2 outlines the algorithm for diagnosis of a suspected case of TRALI.

Differential diagnosis of TRALI includes, but is not limited to, transfusion-related circulatory overload, anaphylactoid reaction to transfusate, bacterial contamination of transfusate, and hemolytic transfusion reaction [8].

There are no specific investigations since there is no specific abnormality associated with TRALI. However, investigations are required to rule out other possibilities of a transfusion-related reaction. Thus in regular clinical practice, TRALI is a diagnosis by exclusion because it has no specific symptoms, signs or investigations. The only routine laboratory parameter that has been associated, albeit infrequently, with TRALI is leucopenia [8].

Laboratory findings for TRALI are inconsistent and include acute transient neutropenia, presence of matching leukocyte antigen-antibody in the donor and recipient, and increased neutrophil priming activity in transfused blood [6,8].

Clinically useful tests to differentiate between cardiogenic pulmonary edema and TRALI include B-type natriuretic peptide (BNP) and determination of the protein concentration in the pulmonary edema fluid and serum.

In patients with an endotracheal tube in place, high protein concentration found in edema fluid sampled within the first hour of intubation may help differentiate TRALI from fluid overload and cardiogenic pulmonary edema [1]. Edema fluid/plasma protein ratio measured by taking matched samples of edema fluid from an endotracheal tube and plasma sample for protein measurements can be diagnostic of increased permeability pulmonary edema. In hydrostatic pulmonary edema, this ratio is <0.65, while it is >0.75 with increased permeability pulmonary edema [2]. This method is valid only for undiluted pulmonary edema fluid, not BAL.

BNP is a biochemical marker of volume and pressure overload [14]. It is secreted from the ventricles in response to changes in pressure when heart failure develops. TRALI is more likely if the BNP is less than 150 pg/ml; however, BNP of more than 250 pg/ml is indicative of congestive heart failure.

A scheme of proposed investigations in a case of any adverse clinical event temporally associated with blood or blood product transfusion is given in Table 2. In the majority, TRALI is a self-limiting condition that is believed to have a better short-term prognosis than other causes of acute lung injury [1].

**Table 2:** Proposed scheme of investigations for an adverse event following a transfusion

S. No.	Investigation	Comment
1.	ABO typing	To confirm type

2.	Direct anti-globulin test	To exclude cross-match incompatibility

3.	Complete blood counts	Transient neutropenia is seen with TRALI

4.	Peripheral blood film	Hemolytic cells may be seen in cross-match reaction

5.	Chest X-ray	Needed to exclude pulmonary edema, pneumonia, other reasons for hypoxia

6.	Blood cultures	Bacterial contamination is a differential diagnosis

7.	Anti-body panel	Includes anti HLA-1 & HLA-2, anti granulocyte, anti monocyte, anti IgA

8.	D-dimer/FDP	To evaluate for deep vein thrombosis

9.	ECHO	For cardiac function status and fluid overload

10.	ECG/Cardiac enzymes	For cardiac function status (to exclude myocardial infarction)

11.	Undiluted pulmonary edema fluid	From endotracheal tube if present – can be diagnostic if fluid to serum protein ratio is >0.75

12.	BNP	Helps to rule out overload in difficult cases (TRALI more likely if BNP < 150 pg/ml)

FDP: fibrin degradation products; ECHO: echocardiogram; BNP: B type natriuretic peptide

Management of TRALI is supportive, as it is for any patient with permeability pulmonary edema, and often includes ventilatory support. Most patients recover with supportive care although approximately two-thirds of patients will require mechanical ventilation with a hospital mortality of 5 to 15% [8].

Patients with TRALI are often normotensive to hypotensive with normal or low filling pressures [1]. Too often, hypoxia that develops after transfusion therapy is ascribed to volume overload, and diuretics are empirically administered. Mild to moderate cases of TRALI may be misdiagnosed as volume overload, and the chance to make a diagnosis of TRALI, and possibly prevent future cases, is lost. There is evidence that diuretics may be contraindicated, and intravenous fluids should be administered as necessary, titrated to achieve mean arterial pressures of 60 mmHg with appropriate urine output [14]. Invasive hemodynamic monitoring may be necessary in especially severe cases to guide fluid management [1,15]. For mild TRALI cases, supplemental oxygen and supportive care may be sufficient for treatment. For the more severe cases, intravenous fluids and mechanical ventilation are necessary. Lung protective (low tidal volume with low plateau pressures) ventilatory strategies should be employed when ventilating TRALI patients [15].

There are reports, but no prospective randomized trials, of use of glucocorticoids in the management of TRALI and, at present, their role in this setting remains unsettled [11].

Recurrent TRALI cases have been described [16], so indications for future transfusions in a TRALI patient should be scrutinized and the patient monitored carefully to determine if a transfusion is needed at all.

## Conclusion

TRALI is emerging as one of the most common causes of self-limiting, yet potentially life-threatening, transfusion associated morbidity, and diagnosis requires a high degree of suspicion. It is based primarily on exclusion of other causes and the supportive clinical picture in the setting of a temporal relation to blood product transfusates. Correct diagnosis is important as diuretics are contraindicated and hypovolemia needs to be corrected. Treatment is mainly supportive, with a significantly better prognosis compared to other causes of acute lung injury.

## Consent

Written informed consent was received from the patient for publication of this case report and any accompanying images. A copy of the written consent is available for review by the Editor-in-Chief of this journal.

## Competing interests

The authors declare that they have no competing interests.

## Authors' contributions

SS contributed to the concept, design and definition of intellectual content along with the literature search, data acquisition and analysis and manuscript preparation. AGH was instrumental in the concept, design, definition of intellectual content, data acquisition and analysis and manuscript preparation, editing and review. DKV defined the concept and intellectual content, helped in data analysis and manuscript editing and review. JP contributed to the design, intellectual content, literature search and acquisition, and manuscript editing. All authors have participated sufficiently in the work to take public responsibility for appropriate portions of the content.
